# Brainstem hemorrhage secondary to retro-odontoid synovial cyst compression: an imaging diagnosis of a stroke mimic

**DOI:** 10.12701/jyms.2026.43.37

**Published:** 2026-06-01

**Authors:** Marina Severo Moraes Michel, Márcio Luís Duarte, Gustavo Andreis, Luiz Fellipe Curvelo Ciraulo Santos, Vanessa Caroline Almeida Dias

**Affiliations:** 1 Department of Medicine, Universidade Federal de Santa Maria, Santa Maria, Brazil; 2 Department of Radiology, Campus Guarujá, Universidade de Ribeirão Preto, Guarujá, Brazil; 3 Department of Radiology, Diagnósticos da América S.A. (DASA), São Paulo, Brazil; 4 Instituto de Ensino e Pesquisa DASA, IEPD, São Paulo, Brazil; 5 Department of Medicine, Prevent Senior, São Paulo, Brazil

A 72-year-old man with a history of hypertension and atrial fibrillation presented with sudden-onset left hemiparesis, dysarthria, and ataxia after temporary discontinuation of rivaroxaban for a dental procedure. On admission, his blood pressure was 210/74 mmHg. Physical examination revealed decreased consciousness, without any signs of systemic infection.

Initial contrast-enhanced computed tomography (CT) demonstrated a well-defined cystic lesion measuring 3.3×1.4×1.4 cm at the anterior craniocervical junction, located between the clivus and odontoid process, causing posterior displacement and compression of the medulla oblongata (Fig. 1A, 1B).

Three days after the initial CT scan, the patient developed progressive neurological deterioration requiring orotracheal intubation. This clinical worsening prompted further evaluation with magnetic resonance imaging (MRI). MRI performed on day 7, following neurological deterioration, revealed a larger extra-axial cystic lesion (3.8×2.6 cm) centered at the atlanto-odontoid joint, with severe bulbar compression and associated vasogenic edema (Fig. 1C, 1D). Follow-up MRI on day 9 demonstrated increased edema and new T1 hyperintensity within the medulla, consistent with a secondary intra-axial hemorrhage (Fig. 1E, 1F). The patient was managed in the intensive care unit with strict blood pressure control, ventilatory support, and discontinuation of anticoagulant and antiplatelet therapy. During hospitalization, the patient remained dependent on mechanical ventilation, and a subsequent attempt at extubation was unsuccessful. Management included continuous neurological monitoring and supportive care. Follow-up neurological examination showed partial clinical improvement, although the diplopia, impaired visual fixation, dizziness, and imbalance persisted. Examination revealed spontaneous upbeat vertical nystagmus and residual axial and appendicular ataxia. Follow-up MRI, which was performed 4 months after the MRI that demonstrated secondary intra-axial hemorrhage, showed a marked reduction in bulbar edema, with residual focal hypointensity consistent with hemosiderin deposition (Fig. 2).

Synovial cysts and retro-odontoid pseudotumors are rare extra-axial lesions arising from degenerative or inflammatory changes in the atlantoaxial joint [1-4]. Although typically indolent, they may cause severe brainstem compression when they are large or rapidly progressive [2-5]. In such cases, sustained brainstem compression may be associated with blood–brain barrier disruption, vasogenic edema, and, rarely, intra-axial hemorrhage [3,5]. Additional mechanisms including venous congestion and perforator compromise may also contribute to this phenomenon. However, a direct causal relationship cannot be established, and factors such as severe hypertension and anticoagulation changes may play a role.

On imaging, these lesions appear as cystic or heterogeneous masses centered at the atlantoaxial joint. In the differential diagnosis, retro-odontoid pannus and degenerative pseudotumors may show more solid or enhancing components, whereas arachnoid cysts are typically not centered at the atlanto-odontoid joint. In addition, cystic neoplasms are less likely in the absence of solid enhancement. In this patient, the imaging features suggested a synovial cyst. MRI is essential for assessing lesion characteristics, brainstem compression, and associated parenchymal changes [3,5].

Management depends on the severity of symptoms. While patients who are stable can be treated conservatively, those with significant brainstem compression or neurological deterioration may require neurosurgical intervention, including decompression or stabilization.

Clinically, differentiation from brainstem stroke is essential. Although vascular events are typically sudden and maximal at onset, compressive lesions may show progressive deterioration. Early impairment of consciousness may also suggest an alternative etiology.

This study has some limitations. Notably, no surgical or histopathological confirmation was obtained, and the diagnosis was based on imaging findings and clinical correlations. However, the characteristic location, morphology, and imaging features of the lesion strongly supported a presumptive diagnosis of a retro-odontoid synovial cyst.

To avoid potential misdiagnosis and guide appropriate management, radiologists should consider retro-odontoid synovial cysts in the differential diagnosis of craniocervical junction masses that are associated with brainstem compression, particularly when imaging demonstrates cystic morphology without features suggestive of vascular pathology [2,5].

## Figures and Tables

**Fig. 1.**
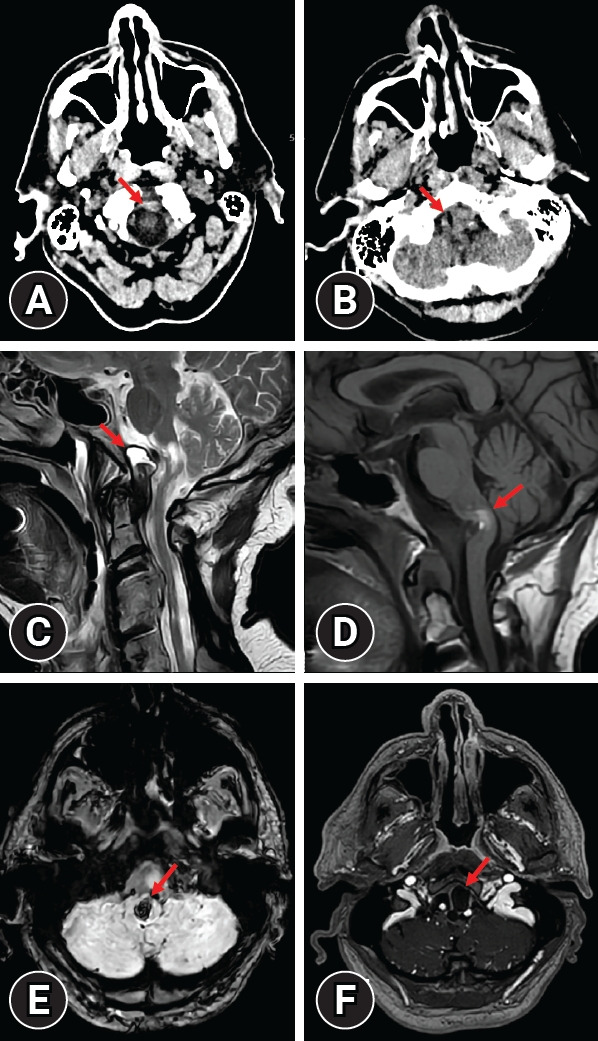
(A, B) Axial non-contrast computed tomography images demonstrate a well-defined extra-axial cystic lesion at the craniocervical junction, centered between the clivus and the odontoid process (arrows), causing posterior displacement and marked ventral compression of the medulla oblongata, without evidence of acute intracranial hemorrhage. (C) Sagittal T2-weighted magnetic resonance imaging (MRI) shows a heterogeneous hyperintense cystic lesion arising from the atlanto-odontoid joint (arrow), with superior extension into the pre-bulbar cistern. (D) Sagittal non-contrast T1-weighted MRI demonstrates mass effect with anterior indentation and deformation of the ventral aspect of the medulla (arrow), extending into the pre-pontine cistern. (E) Axial non-contrast T1-weighted MRI reveals intrinsic hyperintense signals within the medulla oblongata (arrow), consistent with subacute intra-axial hemorrhage, associated with surrounding vasogenic edema. (F) Axial post-contrast T1-weighted MRI with subtraction shows no internal solid enhancement in the lesion (arrow), supporting the cystic and non-neoplastic nature of the lesion.

**Fig. 2.**
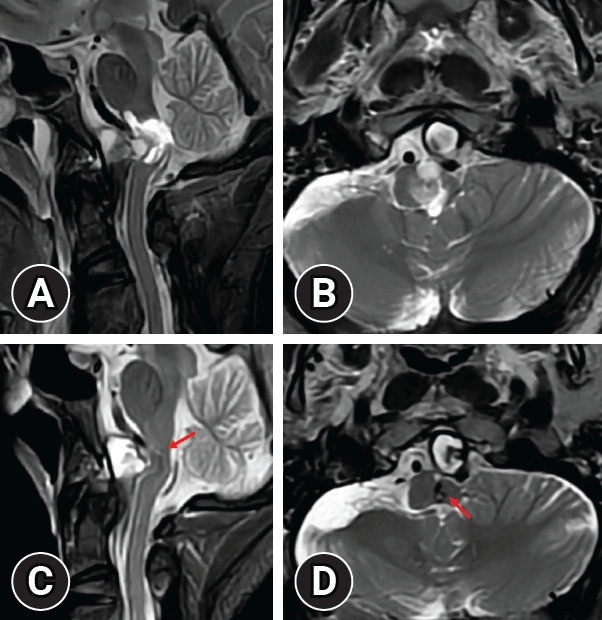
(A, C) Sagittal T2 STIR and (B, D) axial T2-weighted magnetic resonance images. Baseline images obtained at diagnosis (A, B) demonstrate severe bulbar compression by the retro-odontoid cystic lesion. Follow-up images obtained 4 months later (C, D) demonstrate marked reduction in the bulbar edema. Residual focal hypointense signal within the medulla oblongata (arrows) is consistent with hemosiderin deposition related to prior intra-axial hemorrhage. STIR, short tau inversion recovery.

## References

[1] Spennato P, Palmiero C, Cascone D, De Martino L, Bruno G, Cinalli G (2025). Acute ischemic stroke caused by compression of the artery of Percheron by arachnoid cyst. Childs Nerv Syst.

[2] Pereira-Filho A, Faria M, Bleil C, Kraemer JL (2008). Brainstem compression syndrome caused by vertebrobasilar dolichoectasia. Arq Neuropsiquiatr.

[3] Hong JM, Kim DS, Kim M (2021). Hemorrhagic transformation after ischemic stroke. Front Neurol.

[4] Goel A (2015). Retro-odontoid mass: an evidence of craniovertebral instability. J Craniovertebr Junction Spine.

[5] Kovacs KB, Bencs V, Hudak L, Olah L, Csiba L (2023). Hemorrhagic transformation of ischemic strokes. Int J Mol Sci.

